# Supplementary material for: Changes of Physicochemical, Bioactive Compounds and Antioxidant Capacity during the Brewing Process of Zhenjiang Aromatic Vinegar
Source: Molecules. 2019 Oct 31;24(21):3935. doi: 10.3390/molecules24213935 (PMC6864686; doi:10.3390/molecules24213935)
Supplement: Supplementary file 1 [file molecules-24-03935-s001.pdf]

Table S1. Browning index of ZAV samples during the aging process[1, 2].

Aging process (AP) samples	Browning index (OD 420 nm)
0 year	0.26 ± 0.01 a
2 years	0.53 ± 0.01 b
4 years	0.67 ± 0.01 c
5 years	0.71 ± 0.03 d
6 years	0.83 ± 0.03 f
7 years	0.74 ± 0.06 e

[1] Data are presented as mean ± S.D. (n = 3). [2] Significant differences are evaluated using the Duncan Multiple comparison Test. Different letters in the column presents statistically significant differences ($p < 0.05$).
